# 

**Published:** 1859-10

**Authors:** 


### CONTENTS.

### *ORIGINAL COMMUNICATIONS.*

PAGE.
ARTICLE I.—Two Obstetrical Cases in Dwarfs. By A. J. SHAFFER, M. D., of Lawrenceville, Georgia,.......... 65
ARTICLE II.—Odontalgia. By J. P. H. BROWN, Dentist, Atlantn, Geo.,... 71
ARTICLE III.—Bile—Its Offices in Health, Abnormal Effects, &c. ; a These presented to the Faculty of the Atlanta Medical College. By J. E. BLACKSHEAR, Marietta, Geo.,.......... 75
ARTICLE IV.—Quackery and its Cure. By A. G. THOMAS, A. M., M.D. of Atlanta, Georgia,.......... 82

### *SELECTIONS.*

An Inquiry into the Character of Green and Malænial Discharges from the Bowels,.......... 92
Ligature of the Common Iliac Artery for Aneurism—Use of Silver Ligature, 102
Who discovered Anæsthesia ?.......... 107
Demoralization a Source of Disease,.......... 110
Labor, with the Hymen unbroken,.......... 111
Replacement of nn Extracted Tooth,.......... 112

### *EDITORIAL AND MISCELLANEOUS.*

Atlanta Medical College Commencement,.......... 113
Winter Course of Lectures,.......... 114
Dr. Hillyer's Address,.......... 115
Medical Department of the University of the Pacific,.......... 115
Atlanta Mineral Spring,.......... 116
Typhoid Fever,.......... 117
Valuable Surgical Discovery,.......... 121
Easy method of Extracting Eoreign Bodies from the Eye-lids.......... 123
Alleged Abortion...Inquest by the Coroner,.......... 124
New Mode of Relieving Retention of Urine,.......... 124
Remarkable Luxation of the Eye,.......... 126
Case of Needle-swallowing,.......... 126
Detection of Pregnancy by Ergot,.......... 127
Scarlatina after Parturition,.......... 127
Meteorological Table,.......... 128
List of Payments,.......... 128

